# Report on the Malignant Disease, Which Prevailed in the City of New-York, in the Autumn of 1805: *Addressed to the Governor of the State of New-York*
*The City of New-York lies in N. lat. 40, 42, 8; W. long. 74, 9, 45; at the confluence of the river Hudson and Long-Island sound or the East river; and on the southern and narrow extremity of Manhatton-Island, which is about 15 miles in length, and from one to two in breadth. The scite of the City, as it originally stood, was very irregular, being broken into hills and declivities, and indented with small rivulets or creeks, skirted with marsh. Many of the hills are levelled; but the marshy grounds, though covered with houses and pavement, are still low and moist. The City is about 27 miles from the ocean, and is washed on both sides with water of great depth, whose current is very rapid, whose tide ebbs and flows about 6 feet, and which is nearly as salt as that of the neighbouring sea. On both sides of the City considerable encroachments have been made on the water by artificial ground, the whole extent of which may be computed at not less than 132 acres. Of this, 90 acres lie along the East river, and 42 along the Hudson. The portion of it on the East river forms that part of the City where malignant fevers have always first become epidemic and chiefly prevailed. The wharfs and docks are constructed of logs and loose stones. All the fresh water used by the inhabitants is procured from wells within the City, and is now become extremely impure. The population of New-York may be estimated at about 80,000.


**Published:** 1807-02

**Authors:** Edward Miller

**Affiliations:** Resident Physican for the City of New-York


### Medical and Physical Journal.

VOL. XVII.] FEBRUARY, 1807. [NO. 96.

*Printed for R. PHILLIPS, by W. Thorne, Red Lion Court, Fleet Street, London.*

### REPORT ON THE MALIGNANT DISEASE, WHICH PREVAILED IN THE CITY OF NEW-YORK,* IN THE AUTUMN OF 1805: *Addressed to the Governor of the State of New-York.* By EDWARD MILLER, M. D. *Resident Physican for the City of New-York.*

THE Malignant Disease which prevailed in this city, for a considerable part of last autumn, having ceased about the beginning of November, it becomes my duty to lay before your Excellency such an account of it as my official situation has enabled me to collect. I undertake this task with the more readiness, and shall examine the subject with the more attention, as this disease has lately acquired great additional importance from the frequency of its recurrence, the extent of its ravages, and the new

* The City of New-York lies in N. lat. 40, 42, 8; W. long. 74, 9, 45; at the confluence of the river Hudson and Long-Island sound or the East river; and on the southern and narrow extremity of Manhatton-Island, which is about 15 miles in length, and from one to two in breadth. The scite of the City, as it originally stood, was very irregular, being broken into hills and declivities, and indented with small rivulets or creeks, skirted with marsh. Many of the hills are levelled; but the marshy grounds, though covered with houses and pavement, are still low and moist. The City is about 27 miles from the ocean, and is washed on both sides with water of great depth, whose current is very rapid, whose tide ebbs and flows about 6 feet, and which is nearly as salt as that of the neighbouring sea. On both sides of the City considerable encroachments have been made on the water by artificial ground, the whole extent of which may be computed at not less than 132 acres. Of this, 90 acres lie along the East river, and 42 along the Hudson. The portion of it on the East river forms that part of the City where malignant fevers have always first become epidemic and chiefly prevailed. The wharfs and docks are constructed of logs and loose stones. All the fresh water used by the inhabitants is procured from wells within the City, and is now become extremely impure. The population of New-York may be estimated at about 80,000.

and alarming points of view in which it is now considered by the nations of Europe. The embarrassments of our commerce on this account, in foreign ports, have been increasing for several years; they are already become oppressively great; they are likely hereafter to become still greater: and nothing but a thorough investigation of the subject, and the adoption of a wise and mature system of measures, will be sufficient to ascertain and set in operation any adequate means of relief.

In former seasons, it has been usual to observe sporadic cases of this disease for several weeks before the commencement of the epidemic. This was remarkably verified in the late season ; and such cases deserve the more attention as they furnish the best means of calculating the probability of approaching pestilence. Accordingly, one case of a decidedly malignant character was observed in the month of June; several took place in July; a still greater number in August; and at the beginning of September, they had become so numerous as to ascertain the existence of the epidemic. Throughout September and October, the disease continued to prevail with more or less severity, according to the fluctuating states of the weather; but towards the close of the latter month, the coldness of the season had evidently checked its progress; and at the beginning of November, the city was nearly restored to usual health.

During the early period of the epidemic, nearly all the cases took place on the eastern side of the city, in Front, Water, and Pearl streets, and principally below Burling slip. They afterwards became more generally diffused. About the 20th of September, they began to prevail near the North River.* On the whole, the low grounds on the margin of the two rivers certainly produced a chief part of the cases. The number of deaths of the disease in the city, amounted to about 200; those at Bellevue Hospital to 52; and those at the Marine Hospital, sent from the city, to 28. The number of cases of malignant fever reported to the

* A similar extension of the disease, in the epidemic of 1803, was ascribed by many to the removal of shipping from the East to the North river. As no such removal to that part of the city took place in the late season, it is necessary to explain the fact in some other way. This becomes very easy, when it is recollected that the *made ground* on the North river is much less extensive, and the materials composing it much less foul and corrupt, than that on the East river. The miasmata come to maturity on the one side two or three weeks sooner than on the other.

the Board of Health amounted to about 600. It is proper, likewise, in estimating the extent of the epidemic, to notice an unascertained number, probably about 40, who after their flight from the city, died in various parts of the country.

The source of this disease forms a most interesting subject of inquiry; on the success of which must depend all rational and adequate means of preventing and eradicating the evil. After a long and careful investigation of the subject, I cannot hesitate to conclude, that *a pernicious exhalation or vapour floating in the atmosphere,* is the primary and essential cause of this disease. In order to produce this vapour, it is necessary that there should be a concurrence of heat, moisture, and a quantity of decaying animal and vegetable matter. It is therefore exhaled by heat from low and moist grounds, overspread with the corrupting offals of animal and vegetable substances, from such substances collected in large masses, or from any place where the process of putrefaction is going on to considerable extent. This exhalation likewise abounds more in some situations than in others. It is more frequently and copiously produced, and more highly concentrated, in warm and tropical countries than in high latitudes and frozen regions. It prevails and exerts its pernicious influence peculiarly in certain climates, seasons, and local situations. It is generated more in summer and operates more powerfully in autumn than in the other seasons of the year; and it is uniformly more frequent and virulent in sea-port towns, in situations along sea-coasts, in plains, and near rivers, lakes, marshes and swamps, or wherever stagnant waters are found, than in the interior, high and mountainous districts of the country. It is undoubtedly one of the most universal causes of disease in nature. However diversified in quantity or virulence by local circumstances, or by varieties of climate, season, or the condition of society, its effects in one degree or another are nearly co-extensive with the habitable parts of the globe.

While the noxious exhalation just described, when existing in a high degree of virulence, is considered as forming the primary aud essential cause of our disease; it is proper, in order to be well understood, to notice the operation of certain *secondary* or *exciting causes.* These are, exposure to heat, fatigue, cold, intemperance, fear, anxiety, &c. some of which are, in general, immediately instrumental in bringing on the disease in persons predisposed to it by the agency of the atmospheric poison. The noxiousness of this poison, by avoiding exciting causes, may often be long borne without falling into illness; and hence the operation of exciting causes in suddenly producing the disease is often so striking as to lead many entirely to overlook the effect of the principal agent.

The sources of pernicious exhalation in this city are unhappily very numerous and difficult to correct. Some of them are evils of such magnitude and extent, that it requires resolution to consider them, and not to relinquish, in despair, the work of reformation. The mode of constructing our wharfs and slips would almost induce the belief that they had been designed for repositories of filth and nurseries of disease. The *made ground* on the East river is pregnant with almost annual pestilence; it is now become enormously extensive; it was originally composed of the most corrupt materials; from its relation to the river, and the condition of the wharfs and slips, it must constantly remain moist; from its surface being nearly level, it receives and retains the collected filth washed down from the higher grounds; and besides all this, the offensive and putrid matter, which a crowded population must necessarily deposit, and which already underlays a great proportion of this part of the city, incessantly augments the mass of corruption. Can it possibly excite surprize, that the scorching heat of summer, operating on the complicated pollution of this ground, formed of an aggregate of nuisances, and still the receptacle of numberless others, should exhale poison and death into the atmosphere which stagnates over its surface?

As the materials of putrefaction and the degrees of heat, in a large city, greatly exceed what is found in the adjacent country; so the diseases arising under such circumstances must be proportionably more malignant. The pestilential fevers of our city differ only in grade from the bilious and remittent fevers of the country. They prevail in the same climates; they come on at the same season of the year; they are chiefly disposed to attack persons of the same constitution; they commit their ravages on the same organs of the body, and produce symptoms differing only in degree; and they decline and disappear at the same season and under the same circumstances. In the city we often see in the same family and under equal circumstances of exposure, the malignant forms of pestilence and the mild forms of remittent fever; and in the country, while while the great mass of cases are usually mild, we occasionally meet with some which exhibit the violent attack, the intense malignity, and the rapid dissolution, which more frequently mark the pestilential fevers of the city.

Besides the points of analogy just mentioned, there is another equally or perhaps more remarkable. The remittent fever of the country, and the malignant fevers (denominated *yellow)* of our cities, have a similar irregularity which generally characterizes them, and leads strongly to the inference of the similarity of their origin. In the districts of the country where remittent fevers prevail, and in the cities which produce malignant fevers, we find these diseases, in seasons apparently similar, and even in the same season, often exhibiting a singular local unsteadiness in their appearance, extent, and violence. In the operation of the causes which produce them, there is something remarkably contingent and desultory. Remittent fevers will prevail sometimes in one district of a low country and sometimes in another; while the whole extent of these different districts seems to be equally liable to the disease, and no adequate cause can be assigned for the visitation of the one, and the escape of the other. In like manner, some of our cities are invaded by pestilence, in unfavorable seasons; while others, apparently just as liable to be invaded, escape.

For these reasons, as well as many others which my limits will not allow me to state, I conclude that our late epidemic, and all the preceding similar ones, have been of domestic origin, and, of course, nearly related to the remittent bilious fevers of the country.

From this simple and consistent view of the subject, the attention of some has been unfortunately drawn aside by the mistaken opinions of the *importation of the disease from abroad*, and *the propagation of it by contagion*.

1. As the question of contagion, in this disease, is important and fundamental, and as the affirmative has been asserted with much confidence, it becomes necessary to consider this point with great attention.

But, before proceeding to offer reasons in detail against the contagiousness of yellow fever, it is proper to premise some general observations on the subject.

A contagious disease is distinguished from all others by the property of generating or secreting a matter, which, applied by contact, or inhaled with the air by near approach to the sick, or to inanimate substances charged with their effluvia, successively reproduces the same disease. As this contagious matter is secreted by a morbid action of vessels, or a peculiar process of the disease, forming a specific and essential part of its character, it must always be generated when such disease exists; and being generated, and then duly applied or inhaled, its action is altogether independent of external circumstances, such as the state of the air, &c. and must always take effect, unless there be something in the condition of persons exposed to it, which renders them unsusceptible of the impression. This unsusceptibility, depending upon peculiar and unusual circumstances, (except in the diseases which attack the same person but once) must of course be extremely rare. The small-pox affords an example of this operation of contagion. If forty persons, who have never undergone small-pox, be closely exposed to the effluvia of a number of patients lying ill of that disease in the ward of a small-pox hospital, thirty-nine certainly, and probably the whole number, will be infected. This is an example of a contagious distemper. The contagious matter is the constant and universal product of the disease; and when produced, it generally reproduces itself in such as receive it; provided they have not been (in the case of small-pox) previously subjected to its action. The principle of unsusceptibility cannot reside in the surrounding air, but is to be sought for in the body that resists the contagion. There are no facts to prove that pure atmospheric air is a neutralizer or destroyer of contagion; every day presents instances of the reverse; and when diffused through an extensive space, air renders it harmless, not by immediately decomposing, but by diluting and dissipating it. On the other hand, none of the truly contagious diseases derive any additional force from impure air; for the greater contagiousness of confined air in cases of this sort, arises merely from the concentration of a greater quantity of contagious matter within a small space. The application of these principles to the subject in question will presently be seen.

It is proper likewise to premise, that the attack of many persons in the same neighbourhood, or even of whole families, by a reigning disease, affords no proof of contagion;* for the intermittent and remittent bilious fevers of the

* In the course of the autumn, about five years ago, ninety-eight out of a hundred of the labourers employed at the Onondaga Salt Works, in this State, the country, which undoubtedly are not propagated by contagion, often attack families and neighbourhoods so generally as scarcely to leave healthy persons in sufficient number to attend the sick. The want of due discrimination between the effects of an *impure atmosphere* and of *contagion*, is one of the most lamentable deficiencies in the history of diseases.†

The agency of contagion in the propagation of our malignant disease is rejected for the following reasons.

1. No relation is observed between the source of the pretended contagion, and the spreading of the disease to individuals or families; nor was there ever any foundation to attempt progressively to trace the propagation of it to any number of persons, from the first case, or from any single point of infection. If the first ten or twenty cases, which occur in any season, be strictly scrutinized, most of them are found, in their origin, to be distinct and independant of one another. Instead of pervading families, or creeping slowly from one neighbourhood to another, in the track of infection, as is invariably the case with contagious distempers, this disease is found scattered at distant and unconnected points, and cases start up singly in situations

State, were attacked with bilious fever. The two who escaped, probably owed their exemption to extensive ulcers with which they happened, at that time, to be affected. That situation is unusually sickly in the summer and autumn; and a large proportion of the cases of fever which occur there, become malignant and fatal. By the death of several persons, within a few years, who held the office of Superintendant of the Works, and who fell victims to this malignant fever in close succession, that station is now justly regarded by the people of the neighbouring districts, as extremely hazardous.

† Some epidemic diseases, such as small-pox, &c. are considered, by universal consent, as *contagious;* others, such as bilious remittent fevers, &c. are considered as *non-contagious.* It becomes, therefore, extremely interesting to ascertain the criteria by which this discrimination among epidemic distempers may be clearly and promptly made. The want of precision on this point has produced much collision of opinion and much absurdity of conduct among physicians and others. The most obvious criterion, and that which is most generally recognized by the common sense of mankind, is the effect of personal intercourse between the sick and the well. Where a disease is truly contagious, this intercourse cannot fail to disclose the danger, which was long ago correctly stated in poetical language;

"Quo propior quisque est, servitque fidelius ægro,

"In partem lethi citius venit."

*Ovid Metamorph. lib. 7.*

tions where contagion could neither be traced nor suspected.* The proportion of single cases in the midst of families is always great; and the instances of any large proportion of families being attacked were comparatively very rare in our late epidemic. It appears from the records of this epidemic, that there were thirty-one streets of the city, most of which continued to be crowded with inhabitants, in which only a single case in each occurred; and in the mass of six hundred cases, reported to the Board of Health, there were only thirty-five houses in which more than a single case was found. If the number of deaths should be supposed to afford better ground of calculation, it will be found that there were forty streets, and those generally crowded throughout the season, in which only one death in each took place; not more than three died in one house, of which there were only two instances; and, during the whole epidemic, there were only twelve instances of two persons dying in one house.† The great mass of persons attacked with the disease, consisted of such as never had approached the sick, or any other assignable source of contagion; and, on the contrary, as will presently appear, great numbers were exposed to close intercourse with the sick, without injury.

In order to explain this scattered, remote, and unconnected occurrence of cases, the advocates of contagion are obliged to resort to the extravagant supposition of the contagion being diffused through an extensive range of atmosphere, or, to use their own singular phrase, of an *inoculation of the atmosphere* by the effluvia of the sick, or of the infected clothing or bedding which were supposed originally

* Not only the dispersion of the cases is adverse to the doctrine of contagion; but the appearance of them in groups in some instances is altogether as much so. Many of the most judicious of our citizens were convinced of the origination of the disease from domestic filth in the year 1798, by the following occurrence. Between twenty and thirty persons, at the commencement of that destructive epidemic, in a small neighbourhood at the lower end of John Street, were suddenly seized with the disease in one night, in consequence of a blast of putrid exhalations from the sewer of Burling-slip. The persons attacked were only such as lived directly to the leeward of this blast from the sewer; while others, close in the vicinity, but not exposed to this current, entirely escaped.

† From these reports to the Board of Health, it results that upwards of five hundred, out of six hundred cases of malignant fever which occured, were single in the respective families; and that more than three-fourths of the deaths which took place in the city, were likewise single in the respective families in which they occurred.

originally to have introduced the contagion. It is scarcely necessary to observe, that this is a new and unheard of doctrine, utterly unknown and repugnant to all the principles and laws of the communication of contagion, which have been sanctioned by the experience of ages, and entirely subversive of all the hopes the contagionists themselves can repose on the separation of the sick from the well, or on the most rigid regulations of quarantine. This doctrine is likewise inconsistent with itself. If contagion from a single source can extend itself so far, what would become of the inhabitants of the city generally, when, in the progress of the epidemic, cases are so immensely multiplied, and the disease so extremely diffused? If this contagion can exercise such a destructive activity at a distance, after being so much diluted in the air, what must be the effect of approaching near to the source? If a contagion really existed, capable of retaining its virulence, after such extensive diffusion in the atmosphere, it would bid defiance to all the barriers of quarantine, be incoercible by human means, and finally would depopulate the world. Another inconsistency is equally glaring. If this effluvium from a sick body, or from foul cloathing and bedding, can be supposed to vitiate the air to such a distance around, it must, after such extensive diffusion, become light and fugitive, and liable to be blown away by the first breeze. But, how does it happen that this same space of air, after the inhabitants are fled, the sick removed, and houses shut up, continues till a change of season, to be permanently noxious? Nothing can account for this local, stationary, and inexhaustible poison, but the exhalations from the masses of filth and pollution overspreading a large area of ground, forming a vast hot-bed of putrefaction, incessantly teeming with miasmata, and thereby, in despite of currents of air, loading with the seeds of disease every successive portion of atmosphere that sweeps or stagnates over the pestilential surface.

2. The pretended contagion is admitted to produce no effect in our climate, except in particular situations, and at a particular season of the year, when an impure and noxious atmosphere, which ought to be considered as a sufficient cause, is acknowledged to exist. But to consider a disease as contagious, which at the same time exhibits no appearance of that quality but in certain climates; in such climates only in certain places, at such places only at certain seasons, and even at such seasons only after a particular degree of heat and moisture, is undoubtedly to lose lose sight of all the established properties and laws of contagion.

3. It is admitted that the disease does not spread when the sick are removed from the impure air in which it was contracted. By breathing this impure air, without exposure to the effluvia of the sick, persons are every day attacked; while, on the contrary, without breathing it, however exposed to such effluvia, no person is attacked. The conclusion, therefore, is irresistible, that the impure air is the cause.

4. No communication of the disease was ever observed in yellow fever hospitals, situated at a small distance from the cities to which they belong. No exception to this has ever occurred in any of the numerous seasons of this pestilence at our hospital at Bellevue, the marine hospital at Staten Island,* that of Philadelphia, or any other in the United States; provided the malignant air of the city had been avoided. The force of this fact seems never to have been duly considered or appreciated. The numerous retinue of medical attendants, nurses, washerwomen, servants, &c. which belong to a hospital, must be known to every body. How greatly they are all exposed to contagion, if it could be supposed to exist in this case, is equally known. The most malignant degrees of the disease are constantly found in these institutions. The exposure of physicians and their assistants is well understood. The duty of the nurses leads to an incessant and unreserved intercourse with the sick. They pass the greater part of their time, and sleep in the apartments of the sick, the dying, and the dead.† In lifting, undressing, dressing, administering remedies, and many other modes of assistance, they are very often in actual contact, and commonly within a small distance of the patients. They receive and carry away all excrementitious discharges. Several persons

* The two pretended cases of contagion at the Marine Hospital on Staten Island, one in the year 1799 and the other in 1800, were evidently fevers produced by the poison of typhus, modified by the season. Nature is too simple and uniform in her operations to constitute a disease contagious, and yet only so once in a thousand instances.

† The nurses at Bellevue Hospital became so entirely free from all apprehensions of the contagiousness of this disease, that they often slept on the same bed with the sick; and it happened more than once, in the course of the season, that a nurse, overcome with fatigue and want of sleep, threw herself in the night, for a little repose, on the bed of a dying patient, and remained there asleep till the patient was dead, and it became necessary to remove the corpse.

sons are employed in washing the foul clothes and bedding of the sick and the dead. Not only all these have invariably escaped the disease, but likewise all the persons occupied in the removal of the sick from the city to the hospital, who in this service went without reserve into the most pestilential quarters of the town, entered the most filthy apartments, and lifted the sick into their carriages dressed in their foulest clothes, and sinking under the worst degrees of the disease.*

In order to account for these facts, the advocates of contagion contend, that its activity is confined to *impure air*, and that by this alone it can be *conducted* to the objects of its attack. Our hospital at Bellevue, however, is not so constructed as to allow the supposition of great purity of the air; and indeed the state of the land-air in the months of August, September and October, cannot be considered pure in any part of our country. But admitting the highest possible purity of air in these hospitals, the operation of contagion, if it existed there, could not by such means be avoided. When the naked hands of physicians and nurses are in contact with the skin of the patient, scorched with febrile heat, or bedewed with the matter of perspiration, how can pure air be interposed to arrest the passage of contagion? When they inhale, as they often do, the breath and effluvia of the sick, no man can doubt that such air is sufficiently impure to be the *conductor* of contagion, if it really existed. In all contagious diseases, contact and immediate inhalation of the effluvia and breath of the sick, are supposed to constitute the greatest possible exposure; and in such cases, it is plain, the interposition of air, pure or impure, must be equally unavailing to arrest the evil. Yet in these hospitals, persons not only escape this danger, but none was ever known to be infected by it.†

* In order to account for the escape of these persons, which is indeed wonderful, it is proper to state that they all resided during the season at the Alm's-House, an elevated and healthy part of the city, and consequently were only for a short period, at any one time, immersed in the noxious atmosphere.

† In the epidemic of the year 1798, seven persons died of Yellow Fever in our Alm's-House. It was ascertained that they had taken the disease in consequence of going out and breathing the atmospheric poison diffused through the more contaminated districts of the city. Although the house then contained about 800 persons, no communication of contagion took place.

5. The

5. The extinction of the disease by cold weather, is an insuperable objection to the doctrine of its propagation by contagion. That the disease in reality depends upon an atmospheric poison, appears from the fact, that all the means which operate to arrest and destroy it, such as cold, heavy rains and high winds, are merely atmospheric agents. The healthy temperature of the human body is the same in all climates and seasons; and febrile heat is not less in winter than summer. Consequently, the morbid process by which the matter of contagion is generated, is under no controul from atmospheric temperature. Hot climates and seasons are universally held to be unfavourable to the spreading of contagion. The reason is obvious. In warm weather, the doors and windows of the apartments of the sick are kept open, and ventilation is carried to the highest degree. At this season, the effluvia of the body, whether in health or disease, are sooner dissipated, and, of course, can less readily adhere to clothing, bedding, walls, furniture, &c. so as to be retained, and become noxious. In conformity to this, typhus, which is propagated by a poison produced in the clothing, bedding, furniture, &c. of persons living in filthy and crowded apartments, generally prevails and spreads much more in winter, when such apartments are deprived of ventilation. On the contrary, yellow fever, arising from a deleterious principle floating in the atmosphere, and produced by the operation of solar heat upon vegetable and animal filth, ceases to prevail soon after this heat is reduced so low that it can no longer exhale a sufficient quantity of the miasmata of putrefaction. But if this disease depended upon contagion, instead of disappearing at the accession of cold weather, when houses are more closely shut up, it would be then more certainly communicated, and more widely destructive.

6. Yellow fever does not prevail in countries, where the heat is not sufficient to exhale the miasmata of putrefaction, in the requisite quantity and virulence. We hear nothing of this disease in Great Britain, Ireland, or France, though it is well known that persons ill of it, and shipping in which it has recently prevailed, very frequently arrive in their ports. The boarding houses in the sea port towns of these countries, in which seamen arriving from the West Indies are generally lodged, are known to be often extremely filthy and filled with impure air; as appears from the prevalence and ravages of typhus; yet this impure air in those countries cannot *conduct* the contagion of yellow fever.

7. Many

7. Many persons, who had contracted the disease in New-York, died of it at Boston, Albany, and other cities at a distance; many likewise at Greenwich, Brooklyn, and other villages in the neigbourhood. In no instance did these victims of the epidemic communicate contagion. In all these places, the air at that season must have been very *impure*; at Albany and Brooklyn violent remittent fevers were at the same time extremely prevalent; and yet this impurity of the air did not serve as a *conductor* of contagion.

8. Among the early cases of this disease, in the late season, which were, as usual, most virulent, very striking examples of its non-contagiousness were displayed in some of the most crowded quarters of the city. In the beginning of September, a considerable number of sick, who had taken the disease on the eastern side of the city, were removed to the western side; where they died with the most pestilential symptoms. In a house in Cedar-street, where two patients expired under the worst symptoms of this description, the beds* of the deceased, in a very few hours after their death, were occupied by the survivors of the family. Yet in none of these numerous instances was any contagion communicated.

9. The universal exemption of the physicians of New-York, amounting at least to 50 or 60 persons, from the late disease, is also irreconcilable with the doctrine of its contagiousness. I have not heard of any physician in Philadelphia, New-Haven, Providence or Norfolk, suffering illness from their late epidemics. It is known that physicians neither use nor possess antidotes. Their exposure to the breath, effluvia and contact of the sick, was almost incessant from morning till night. They employed no precaution of dress or covering, no fumigation, no means of destroying, neutralizing or obviating, in any manner, the effluvia of their patients. The dissection of bodies dead of Yellow Fever, if contagion had existed, would also have formed another source of danger. Many of the physicians of this city were frequently engaged in the mode of investigating the disease, and minutely examined bodies in a very advanced state

---

* It is proper to observe that, since the first publication of this letter, a contradiction of the statement concerning the beds has been received from one person, and a confirmation of it from another. That particular circumstance is, however, immaterial; as it is admitted on all hands that no contagion arose from either of these malignant cases.

state of putridity. The more happy escape of physicians in the late than in former epidemics, is to be attributed (under the protection of Divine Providence) to their having secured a residence in the higher and safer parts of the town, and to the comparative infrequency of their visits to the districts of envenomed atmosphere; owing to the early desertion of these districts by the chief part of the inhabitants. It is understood, at the same time, that our physicians, in their confidence of the non-contagiousness of the disease, generally passed more time in the apartments of the sick, and were in the habit of making a more deliberate and minute examination of the cases which fell under their care, than in preceding epidemics.*

10. The failure of every attempt to arrest the progress of the disease, by the separation of the sick from the well, is also incompatible with the doctrine of contagion. Besides the numerous ineffectual attempts in this city, the utmost endeavours were used, with the same result, by the Board of Health of Philadelphia, whose members had been purposely selected for this object, from those who embraced the opinion of the importation and contagiousness of the disease. It would be fortunate, indeed, for the purpose of arresting Yellow Fever, if its progress depended upon contagion. This appears from the example of the small-pox, a disease whose contagion is more active, steady and permanent than any other in the world. By a system of quarantine, extremely simple and very little burthensome, this distemper is excluded, or, if introduced, immediately arrested and banished, in Boston and other cities of New-England, where its admission and circulation are prohibited by law.

11. The inconsistency and contradiction which constantly attend the application of the doctrine of contagion in this disease, make it altogether inadmissible. To explain one set of facts, it must infinitely transcend the contagiousness of small-pox; to suit another, it must sink infinitely in the opposite direction. On some occasions, it is more subtle

* The exemption of the nurses from disease, who attended the sick in the city, was also very remarkable. Upwards of sixty persons were employed, by the Board of Health, to perform this duty. Only four of these died; two others only were sick, and recovered. And it appears, upon inquiry, that such as died or were sick, had been stationed in the parts of the city where the atmosphere was known to be most highly charged with the miasmata of putrefaction.

subtle, penetrating and rapid than the electric fluid; on others, more sluggish and dormant than the grossest matter. Contrary to all other noxious substances, it is often more destructive at a distance, than near to its source; for at one time, it cannot reach a single individual among a great number surrounding the bed of the patient, and in frequent contact with his person, while at another, it must strike at the distance of several hundred feet.* THE NOXIOUSNESS OF THE MIASMATA OF PUTREFACTION, EXHALED BY HEAT AND FLOATING IN THE ATMOSPHERE, EXPLAINS ALL THESE FACTS, AND RECONCILES ALL THESE CONTRADICTIONS.

If it were possible to add any thing to the evidence of these irresistible facts, I might subjoin, that Yellow Fever cannot be considered as a contagious disease;—Because, unlike all other contagious diseases, it has no specific character, no definite course of duration, and no appropriate, essential or pathognomonic symptom;—Because, the supposed contagion rarely operates singly, and in general depends upon the co-operation of exciting causes;—and finally, Because, the miasmata which produce this disease are more or less noxious as they are more or less concentrated, a property which does not belong to the specific poisons of small-pox, syphilis, &c.

Under the conviction of these facts, I am compelled to conclude that our malignant disease is the effect of a noxious exhalation floating in the atmosphere, and that it is ABSOLUTELY AND UNIVERSALLY NON-CONTAGIOUS.

For the correctness of the facts on which this conclusion is founded, I appeal to my fellow practitioners and fellow-citizens, who have been witnesses of the disease. For the application of these facts in the deduction of principles and opinions, I appeal to the judgment of physicians in every quarter of the world, where medicine is cultivated as a regular science. And, especially, I would offer this appeal to the liberal and enlightened physicians of Europe, who are sincerely devoted to the cause of truth and professional

---

* While it is admitted that contagion cannot operate in Yellow Fever Hospitals, and while this inactivity of it is ascribed to the absence of impure air; it is, at the same time, gravely asserted by some, that a person going on board of a vessel, lying in a situation where the air is much more pure than it can possibly be at a hospital, even though there exist no sickness on board of such vessel, may still derive contagion from it, and experience all the active and malignant operation of such contagion, notwithstanding this purity of the surrounding atmosphere.

sional improvement, who, on this subject, have heretofore received much incorrect information, and who, as soon as they become convinced of the real state of the question, will, I am confident, exert the influence they so justly possess, in procuring from their respective Governments an abolition of the oppressive and useless restrictions of quarantine, which have been recently imposed on American commerce.

II. The second mistake concerning this malignant disease, which has been impressed on the minds of some of our citizens, is that of its *importation from abroad, and chiefly from the West-Indies.* This opinion is rejected for the following reasons.

1. The non-contagiousness of the disease must entirely destroy the belief of its introduction from abroad. It is impossible to conceive that it can be conveyed across the ocean, and propagated in the cities of the United States, unless it possess the power of successively re-producing itself by communication of contagion from one person to another.

2. If the alleged importation were possible in any case, it might happen at any season of the year. In this active sea-port, Shipping from the West-Indies are very frequently arriving at all seasons; and it is known that yellow fever may be found in those islands at any period of the year, when they are visited by strangers from the higher latitudes: yet the pretended importation is always confined to that period of the summer and autumn, when local and domestic causes, sufficient to produce the disease, are known to exist.

3. If yellow fever could be introduced from abroad, it is impossible to explain its non-appearance in our sea-ports for a long series of years, when no means were used to secure its exclusion. For more than fifty years preceding 1795, no importation of the disease into this city was suspected; and it is indeed uncertain whether, before that year, the opinion of its importation at any period of the eighteenth century, had attracted much attention. The advocates of importation generally assert, that periods of war in the W. Indies are most apt to occasion its introduction into this country. Yet we hear nothing of its being brought to this port during the war of 1756, or that of the American Revolution. In the former of these wars, the mortality attending the successful expeditions against Martinique, Guadaloupe and the Havanna, was almost incredible. Only a very small part of the victorious troops were alive alive three months after their conquest. Equally fatal were the malignant fevers of the West-Indies in the war of the American Revolution. Dr. Hunter* informs us, that of 5,000 troops who took possession of St. Lucie, scarcely a man of the original number remained at the end of one year; although the sword of the enemy had destroyed an inconsiderable amount. The mortality continued as great in subsequent years. From the 1st of May 1780, to the 1st of May 1781, the number of dead was equal to the average strength of the garrison during the year. Of the troops sent from Jamaica upon the expedition against Fort St. Juan, scarcely a man ever returned. During this period, the intercourse between the West-Indies and this port must have been extremely frequent. Doctor Blane† states, that in the course of the war of our Revolution, nearly 18000 sick were landed at New York from the British fleets; that 11 sail of the line arrived here early in September 1780, from the W. Indies; that 26 sail of the line arrived here at the same season in 1782, likewise from the W. Indies; and that from each of these fleets, a great number of sick, afflicted with malignant fevers, were sent to the hospitals at this place. It is also known that a similar fleet arrived here in the beginning of the autumn of the year 1781. During all this period, notwithstanding the ravages of yellow fever in the West-Indies, and the conveyance of so many sick to this port, we hear nothing of the importation of the disease. And yet, at that time, no quarantine-regulations existed.

The contingencies by which yellow fever might have been imported, through the medium of commercial shipping or of naval and military expeditions, if such importation were possible, must very often have occurred in a sea-port like this, where such extensive communication has been so long maintained with the West-Indies. A more frequent introduction of the disease, therefore, according to the doctrine of importation, as now held, must have been inevitable. But as this did not take place for such a length of time, and under circumstances so likely to produce it, we are warranted in the conclusion, that importation is impossible.

On the contrary, as the history of pestilential epidemics, in all ages and countries, demonstrates that they are sub-

* Observations on the Diseases of the Army in Jamaica.

† Observations on the Diseases of Seamen.

ject to frequent revolutions, as to the periods and places of their prevalence, the variety of their symptoms, and the degrees of their malignity; it is much more easy to account for changes in such diseases, as they locally or periodically occur, than for any great diversity or fluctuation in the circumstances or contingencies, which determine their importation from abroad.

4. No importation of this disease, so as to become epidemic, was ever known in any port of Great Britain, Ireland, or France. The vast amount of shipping, as was observed before, which arrive at those ports from the West-Indies, is well known; and, that they often arrive in a very sickly condition, is equally known. The filth and impure air of those ports are admitted on all hands, and the effects of them are experienced in the destructive fevers of a different description which frequently prevail; and yet, for want of the atmospheric heat and other local circumstances requisite in the generation of yellow fever, they are happily strangers to its epidemic prevalence.

5. The appearance of yellow fever in many of the interior parts of the country, inaccessible to foreign contagion, confirms the opinion of its domestic origin, while it entirely invalidates that of its importation. There is not a State in the Union, which has not afforded evidence of the production of the disease, in situations where importation was impracticable. In the course of the late season, a malignant fever, in all essential points the same as our yellow fever, prevailed in many parts of this State, and caused more mortality, in proportion to the population of the district, than took place in this city. There can be no reasonable doubt, that the disease called the *Lake Fever*, in the interior of this State, possesses all the essential attributes of the yellow fever.

6. A comparison of the summer and autumn of the year 1804, with the corresponding seasons in 1805, will go far to shew the dependence of our malignant epidemics on the condition of the atmosphere, and, of course, to overthrow the doctrine of importation. The summer of 1804 was mild and cool, beyond former example, on all the Atlantic coast of the United States, lying to the northward of the Carolinas. In South-Carolina and Georgia, the heat was unusually great. All the Atlantic cities north of the Carolinas, without exception, entirely escaped the epidemic: whereas at Charleston, and in some parts of Georgia, it prevailed with great mortality. On the contrary, the late summer was remarkable for the duration as as well as the intensity of heat, along the whole of our coast. And the consequence was, not only that nearly all the Atlantic cities were visited with pestilence, but, what was still more surprising, that in several of them it made its appearance within forty-eight hours, or nearly, of the same time; an occurrence which cannot be explained on the contingency of importation, and is only to be satisfactorily accounted for from the state of the atmosphere.

7. The occurrence of similar diseases in other parts of the world, under similar circumstances, where contagion introduced from abroad cannot possibly be suspected, is also adverse to the doctrine of importation. In making the circuit of the globe, on the parallels of latitude nearly or exactly corresponding with ours, we pass over countries which, from the earliest records of history, have been frequently visited with the ravages of this scourge. Spain and Italy afford striking examples. The city of Rome, in particular, though its elevated situation is generally salubrious, is annoyed by a marshy spot at the feet of two of its hills, along the margin of the Tiber, which has been sickly and pestilential from the origin of the city. While the streets on the hills, like Broadway and other high grounds in this city, enjoy a salubrious air, the spot of marsh just mentioned, together with a small extent of *made-ground*, (for the madness of *made-ground* has existed at Rome as well as at New-York*), corresponding with our marshy districts and vastly more extended space of made-ground, along the margin of the East River, has produced, from time immemorial, malignant and mortal epidemics. And the medical historian of these facts, (the celebrated Baglivi) expresses his astonishment, that so small a distance, as that intervening between the elevated and depressed portions of ground, should make such a difference in the qualities of the air. As the Tiber is not navigable for

I 2

* *Proofs of this might be adduced from Lancisi and other medical writers of Rome. The following lines are sufficient to establish the fact.*

Hoc, ubi nunc fora sunt, udæ tenuere paludes;

Amne redundatis fossa madebat aquis.

Curtius ille lacus, siccas qui sustinet aras,

Nunc solida est tellus, sed lacus ante fuit.

Quà Velabra solent in Circum ducere pompas,

Nil præter salices cassaque canna fuit.

*Ovid. Fast. Lib. VI.*

for sea-vessels, the importation of their pestilential epidemics at Rome was never suggested.

8. The inefficacy of all the various modifications of quarantine hitherto devised in this country, confirms our disbelief of importation. In this port, as well as in Philadelphia, a rigid system of quarantine has been in operation for many years; and there is no doubt of its having been vigilantly and faithfully executed. Indeed, the experience of quarantine in the United States speaks little in its favour; for though, during the last ten years, it has been scrupulously enforced in several ports, we have heard ten times more of imported contagion and of its ravages, at these very ports, during that short period, than for an hundred years before, when no quarantine was in existence.

9. The entire want of all proof, and even of the least probability of the introduction from abroad of the germ of our late epidemic, gives the last blow to the doctrine of importation. The facts on this subject have been so clearly and minutely detailed by the Health Officer, that it would be superfluous to repeat them here.

The source of mistake, on the subject of importation, consits in not distinguishing *a febrile poison generated by heat und filth in a vessel,* from *contagion taken up in a foreign port, and successively communicated from one person to another.* The construction of vessels disposes them to the collection and retention of filth, and renders cleansing and ventilation extremely difficult. The quality of cargoes and provisions, the inattention of seamen to cleanliness, the crowded manner in which they often live, the unsuspected and inaccessible situations in which corrupting substances may lie concealed, render shipping, independently of the hazards of the element on which they move, the most dangerous of all human habitations. It is no wonder, therefore, that they should become unhealthy, when they pass into warm latitudes, or lie in our harbour in the hot season. In no situation is a malignant fever more apt to originate than in a ship. A vessel that never left our port, or that has remained in it for years, may become foul, and thereby generate and emit a deadly exhalation. Whether malignant fever arise from filth ashore or on shipboard, the principles and process, by which the evil is produced, are still the same. On what ground can a disease be said to be *imported,* which has no other relation to a foreign country, than that of being generated in a vessel which has lately visited that that country? The foreign country, the outward and homeward voyage, are circumstances of no moment in determining the origin and character of the disease; to account for this, we must consider the filth, the moisture and heat, which, concurring to a certain degree, are destructive to man at all times, in all situations and under every condition. And a fever originating under such circumstances can no more be pronounced *imported,* than a fracture of a limb happening at sea can be called an *imported fracture.*

It has been supposed by some, who regard only one aspect of the subject, that the doctrine of importation alone can explain the more frequent recurrence of malignant epidemics for the last ten years. But the difficulty still returns with unabated force; and it remains to explain, why importation has become so much more frequent and easy of late than formerly. If it be thought impracticable to throw light on that peculiar constitution of the air, which determines the prevalence of yellow fever at one time more than another; it is equally impracticable to ascertain the qualities of the air which produce malignant distempers of the throat, the dysentery, and other mortal epidemics, (which are undoubtedly of domestic origin) for a season, or for a term of years, and then allow them to disappear.

It has been said, that the belief of the yellow fever originating in this country, would be destructive to its commerce and prosperity. But if the appeal must be made to interest rather than truth, let us contrast the effects of the two opinions, as they influence our intercourse with foreign nations. By truly describing the disease, and exhibiting the proofs of its local origin and non-contagiousness, we convince foreign nations that it is a misfortune limited to ourselves, that it cannot endanger their safety, and that it only claims their sympathy and regrets. By asserting the importation and contagiousness of it, the evil immediately swells to an indefinite and incalculable extent, and we alarm all nations with the fear of its being, in turn, exported to them. After the experience already gained, neither they nor we can cherish any rational hope of hereafter excluding it, by regulations of quarantine. Our intercourse with the West-Indies, and with all other tropical countries, will be daily extended, and if importation were posssible, the chances of it will be every year progressively multiplied.

On the ground of importation, unless trade be totally forsaken, our situation is hopeless.

In rejecting the doctrine of importation, the benefits of quarantine are by no means intended to be undervalued. The generation of pestilential disease in foul vessels is undeniable; they are certainly a very frequent source of sickness; and all persons concerned in shipping are interested in a careful examination of them. There ought to be some mode of ascertaining whether a vessel may be safely approached by people in business, or whether she may be likely to diffuse pestilential vapours among all who come within their reach. Quarantine is also one of the most humane regulations in favor of seamen, who are confessedly a very useful and necessary class of the community. It interposes between them and the carelessness or cruelty of their commander, and makes it his interest to preserve their lives and health. And while it might be organized so as to answer all these purposes efficaciously, it might also be properly stripped of its useless and burthensome appendages.

If the facts and reasonings, which I have adduced, to prove the non-contagiousness and non-importation of yellow fever, be well founded, it results that our epidemics are local, domestic, and as incapable of exportation to foreign nations, as the bilious fever of the country. It is to be lamented that the reverse of this opinion has made so deep an impression in Europe; and that the Governments of that quarter of the world have suffered themselves so lightly and hastily to embrace doctrines, and legislate on principles, contradicted by all former experience. It is now more than three hundred years since they became acquainted with America. And although the first discoverers of the new world, as well as most succeeding adventurers, have largely shared the effects of the baneful climate of the West Indies; it is only of late that apprehensions have been entertained of importing into Europe the malignant fevers of those islands. The shattered remains of fleets and armies had often returned home to Great Britain and France, in the most sickly state, after encountering all the horrors of yellow fever, without once communicating that disease. But what transmutation can yellow fever undergo in the United States, which renders it exportable to Europe from us, but not directly from the West Indies?

It affords some apology indeed for Europe, that the information formation concerning this subject, upon which they have acted, was derived from our own country. The acts of our Sate Legislatures, the proceedings of our Municipal Bodies and Boards of Health, the proclamations of our Magistrates, and a variety of other public documents, have all a tendency to impress the same opinion. We have held up to foreign nations, an indigenous and local disease, growing up from the infelicities of particular situations, or from neglects of police, and entirely incommunicable from one person to another, as highly contagious, capable of exportation to distant countries, and consequently alarming to the safety of the whole commercial and civilized world. We cannot transplant the disease from this city to the neighbouring villages of Greenwich, Brooklyn, or Newark; and yet it is believed we can convey it three thousand miles across the pure air of the Atlantic. Whole hospitals of patients, labouring under the most malignant forms of the disease, with all the foul apparel, bedding, &c. polluted with the excrementitious discharges and other filth of the sick, the dying and the dead, cannot emit an atom of contagion; and yet we pretend to dread the infectiousness of a sailor's jacket or handkerchief, or even of the cordage and timbers of a vessel. Under the influence of this phantom of contagion, we have instructed the Europeans to enact laws and regulations, sanctioned by the highest penalties, which retard and oppress our commerce, and subject our shipping in their ports to the most grievous detention. To crown the whole of this injury and humiliation, we have instigated them to place the people of the United States, by late extensions of quarantine, on the same footing with the degraded and detestable inhabitants of Barbary, Egypt, Syria, the Archipelago, Constantinople, and other parts of the Turkish dominions. And all this has been done, in defiance of clear and luminous facts, and in the face of long, reiterated and ample experience.

By discarding the bugbear of contagion, the origin and nature of yellow fever will be more truly ascertained; the means of personal safety more generally understood; and the measures necessary to improve the salubrity of the city more vigorously pursued. The public will no longer witness that desertion and misery of the sick, which have too often disgraced society, in every epidemic. The bosom of humanity will no longer be wrung with the sufferings of our fellow-creatures, driven, while under the pressure of this calamity, from every place of shelter, deprived of comfort, and abandoned to their fate, from the false impression of danger in affording them assistance. By telling the community the truth, we shall lessen apprehension and distress, we shall disarm the evil of half its power, and restore the ties of kindred, and of nature.*

It is surely time to investigate this subject with the deepest attention, and to adopt some adequate system of relief. The warning voice of history and experience loudly calls us to make every exertion to deliver our city from nuisances, which threaten to entail the miseries of an annual succession of malignant epidemics. WE LIVE IN THE LATITUDE OF PESTILENCE, AND OUR CLIMATE NOW PERHAPS IS ONLY BEGINNING TO DISPLAY ITS TENDENCY TO PRODUCE THIS TERRIBLE SCOURGE.† The impurities, which time and a police, rather moulded in conformity to the usages of more northern countries than to the exigencies of our own, have been long accumulating, are now annually exposed to the heats of a burning summer, and send forth exhalations of the highest virulence. The examples of similar calamities in many parts of the old continent,

---

* The learned Dr. Hunter, one of the members of the National Board of Health of Great Britain, offers the following argument in support of his opinion of the non-contagiousness of yellow fever. "The strongest proofs of this, in my opinion, were to be met with in private families, where the son, the brother, or the husband, labouring under the worst fevers, were nursed with unremitting assiduity by the mother, the sister, or the wife, who never left the sick either by day or by night, yet without being infected. That such near relations should take upon them the office of a nurse, is matter of the highest commendation in a country, the diseases of which require to be watched with greater care and attention than can be expected from a servant. They are under no fears of the fever being infectious, and I never saw any reason to believe it to be so, either in private families, or in the military hospitals." That Dr. Hunter came to this decision, after a full and mature consideration of the importance of the subject, will appear from the following remarks: "There is hardly any part of the history of a disease, which it is of more consequence to ascertain with accuracy, than its being of an infectious nature, or not. Upon this depends the propriety of the steps that should be taken, either to prevent it, or to root it out. It is productive of great mischief to consider a disease as infectious, that really is not so; it exposes such as labour under it to evils and inconveniencies, which greatly aggravate their sufferings, and often deprive them of the necessary assistance. They are neglected, if not shunned; and at the time they require the greatest care and attention, they have the least."

Observations on the Diseases of the Army in Jamaica, pp. 177, 178.

† To convince the reader of this, it is only necessary to remind him how near the cities of Philadelphia and New-York lie to the parallels on which Rome and Constantinople are situated. It is scarcely requisite to observe, that the ravages of pestilence in these ancient cities have far exceeded any thing which has occurred elsewhere, unless those of Grand Cairo should be supposed to equal them.

tinent, ought long since to have taught us lessons of wisdom. In the city of Rome, time and fatal experience pointed out the necessity of erecting extensive and costly public works, in order to deliver the inhabitants from the horrors of pestilence; and the air of that city was, at several periods of its history, in alternate succession, observed to become pestilential or salubrious, as these public works were suffered to fall into decay, or were repaired and renewed.

The different opinions of the origin of yellow-fever, offer us only the alternative of a more rigid quarantine, or of more vigorous internal measures. Every step of increasing restriction in our system of quarantine, has only served to shew more clearly the domestic origin of the disease. If an entire prohibition of West India trade, or a prohibition during the summer and autumn, were imposed by law, the effect would soon be sufficient to banish every doubt from the mind of the public. How far the advantage of unanimous conviction might be supposed to countervail the burthen of such restrictions for a short period of years, I shall not undertake to decide.

But whatever opinion may be embraced, the present moment is certainly not the time for the indulgence of apathy or inactivity. If the legislature, in their wisdom, should still think that this disease is introduced from abroad, they are bound by the strongest obligations to extend the powers of quarantine, by additional restrictions. The conveniencies of trade are not to be put in competition with the ravages of yellow fever. If it be necessary to resign the freedom of commerce, or to incur the miseries of pestilence, let the former be freely abandoned.

It is likewise my duty, before I conclude, to suggest whatever it may be deemed adviseable to do towards the removal of existing nuisances, and the improvement of the salubrity of the city. This task has been, in some degree, anticipated in my letter to Governor Clinton, after the epidemic in 1803. Unfortunately, some of the requisite measures will demand great expense, and must bring to a test the liberality, enterprize, and public spirit of the city and State. Among the improvements of the most urgent and immediate necessity, I consider the following, to wit, *Water*, obtained from a distant source, of pure quality, and in quantity sufficient to allow a constant, plentiful, and increasing expenditure; *Sewers*, of such number, capacity and construction, as completely to drain all the low and marshy districts, to carry away all filth, and to be constantly constantly washed by a brisk current of water; a new arrangement and construction of wharfs, docks, &c. so as to face the margin of the two rivers with a stone quay, impervious to water; a prohibition to make a single additional foot of artificial ground on either of the rivers; a different modification of privies, which are every day becoming more and more an alarming nuisance, and will soon underlay with filth a large portion of the city; a better plan of paving, more particularly as respects the construction of gutters, &c. the draining of all stagnant waters in the town and neighbourhood, the filling up, levelling and paving all low and depressed lots and places of whatever description; and a prohibition hereafter to inter dead bodies in any part of the city. Many other objects, which would require much minuteness of detail likewise demand attention; and will acquire great additional importance from the rapid progress of building and population.

---

### *APPENDIX.*

Under this title, it is intended to lay before the reader some proofs and illustrations of the principles delivered in the foregoing Report, which could not properly be admitted into the letter itself, and which are too long to have been conveniently subjoined in the form of Notes.

### *On the analogy, as to localities and diseases, between the cities of Rome and New-York.*

It is from the south of Europe, and chiefly from *Spain* and *Italy*, that inquirers into the endemic diseases of the United States may expect to derive the most valuable lessons of time and experience. The writings of the Italian physicians in particular, are full of instruction on this subject; and it is to be lamented, that this instruction has not been more eagerly sought for, and more generally obtained by their American brethren.

By considering the following account of the localities and diseases of *Rome*, given by Baglivi, and comparing them with those of *New-York*, we perceive how exactly like causes will produce like effects, in the old and in the new continent.

" Ut res exemplo fiat clarior, exponemus breviter, quæ nos " Romæ circa aëris temperiem, & medendi methodum quotidiano " usu experimur. Aër Romanus septem collibus, Orbis dominis, " hodie interclusus, naturâ humidus est & gravis; experimento " namque constat, quod si quis paulo longius a frequentia tectorum " processerit, quantam cœli gravitatem atque intemperiem mani- " festo concipiet. Insaluberrimis Austri, Africi atque Euronoti " flatibus obnoxius; ab æstivis caloribus interdum tantopere ex-

" ardescit;

"ardescit, ut mirum non videatur, si Consulibus L. Valerio Portito, "& M. Manlio, Pestilentia orta sit in agro Romano, *ob siccitates & "nimios solis calores*, teste Livio, lib. V. His aliisque de causis "infra dicendis, Incolæ urbis temperamento præditi sunt melan-"cholico, subfusco, & nonnulli subpallido cutis colore, habitu "corporis macilento potius quam pingui; levi de causa capite affi-"ciuntur, & iis morbis potissimum subjacent, quos aëris gravitas "solet producere, sicuti sunt pulmonis vitia, febres malignæ, ca-"chexiæ, pallores vultus, incubus, tabes & consimiles. Porro aër "Romanus squallidus quoque est & insalubris, non quidem omni-"bus in locis, sed iis potissimum, quæ deficientibus ædificiis, pigro "atque immoto aëre sordescunt; multo magis si Tiberi adhærent, "vel convallium instar, montibus obsepiuntur, aut exhalationibus "subjacent quas veteres parietinæ, cryptæ, & antiquorum ædifici-"orum rudera emittunt. Ex quo patet Regionem Circi Maximi, "inter Palatinum atque Aventinum sitam, omnemque illum cam-"pum qui inter Aventinum, ac Tiberim, portamque Ostiensem, "jacet, plane noxium esse & damnabilem. Sed ut rem universim "definiam. Quæcunque loca crebris ædificiis ambiuntur, atque "editiora sunt, in septentrionem atque orientem spectant, & mul-"tum a Tiberi distant, salubriora: Contra, quæ sejuncta sunt, & "remota a frequentibus tectis, situque sunt humili, ac maxime in "convallibus, tum propiora Tiberi, in meridiem atque occasum "spectantia, minus salubriora judicantur: Quibus etiam in locis "(quod sane mirum) brevissimi intervalli discrimine, hic aliquan-"tum salubris existimatur aër: illic contra noxius & damnabilis.

"Insalubritatem hanc urbani aëris, fovet magna ex parte adja-"cens Latium; quod undequaque coronâ montium circumcingitur, "excepto tractu illo, quâ mediterraneum vergit, ubi in planitiem "desinit. Vetus enim Latium desertum ferè hodie est & squalli-"dum; Austri flatibus immediatè objicitur; & variis ejusdem in "locis insaluberrimus aër observatur, utpotè circa Ostiam & Por-"tum, æstivo præsertim tempore; quo quidem si aliquis in præ-"fatis aliisque Latii locis pernoctaverit, & extinde urbem reverta-"tur, corripitur statim maligna febri, quam vulgo, ex mutatione "aëris dicunt; estque febris hæc sui generis, ab aliis febribus, "alias agnoscentibus causas summopere differens, tum in methodo "curativa, tum in symptomatis eandem concomitantibus."

*Georg. Baglivi Oper. Omn.* pag. 157, 158.

Lancisi, in his valuable work *De Noxiis Paludum Effluviis*, confirms the facts stated by Baglivi, and adds many others, which are extremely important. In his account of a malignant epidemic, in the summer and autumn of 1695, which ravaged a particular district of the city of Rome to such a degree as nearly to depopulate it, he traces the disease to its cause in the following words:

"Nemo sane luctuosa funera per id temporis Romæ conspiciens, "fœtoremque in vicis illis persentiens, dubius hæsit, quin causa

"malignarum,

"malignarum, perniciosarumque febrium, quæ publice vagabantur, "fuerit multitudo stagnantium et corruptarum aquarum, tum in "scrobibus pratorum, tum in magna cloaca, atque in fossa potissi- "mum Hadrianæ arcis. Tellus jam erat humida, cum Tiberis "propter magnam vim aquæ bis auctus est; atque idcirco non "solum scrobes, ac fossæ pratorum et Arcis exhauriri non potue- "runt; verum quod maxime aëris insalubritatem inducit, sordes, "quæ pluviis prolutæ everruntur, ac dilabuntur, iis in canalibus "atque in cloacis subsistere coactæ sunt. Simul etiam per humi- "liora Leoninæ civitatis loca exundavit, subterraneasque cellas, "multosque pauperum puteos hic illic contemeravit. Posthæc, "negligentia eorum, qui rebus publicis, atque eidem præsertim "Arci præerant, nullum studium purgandis hisce regionibus ad- "hibitum fuit. Hinc mira hæc proluvies in limosam paludem "sensim intra fossas scrobesque conversa, virescere, jam urgente "æstu, fermentari, computrescere, variaque insecta admittere "cœpit. His vero malis accessit etiam frequens afflatus Vulturni, "austrinorumque ventorum, qui a medio Maio usque ad Septem- "brem identidem recurrentes, non tantum deteriori putredini im- "motarum aquarum, verum faciliori quoque sublimationi ac dela- "tioni malignorum effluviorum non in vicinas duntaxat ædes, sed "etiam usque ad finitimas adversasque regiones, ansam præbue- "runt."

*Lancis, Oper. Var. Tom. 1. p. 189.*

---

### *On the antiquity of the Yellow Fever.*

It has been contended by some, that the yellow fever is a modern disease, and utterly unknown to Europe, except when imported there from America. A slight inspection of the writings of HIPPOCRATES, who flourished upwards of four hundred years before the Christian æra, will be sufficient to prove that he was familiarly acquainted with it, and had observed it under its most malignant and fatal forms.

The two symptoms which are considered as most characteristic of this fever, are *yellowness of skin*, and *black vomiting*. A great number of passages might be adduced to shew that Hippocrates frequently met with these symptoms in the malignant fevers which fell under his care. I shall mention only such as are clear, pointed, and incapable of being mistaken. In the ninth section of his book of Crises, he lays it down as a maxim, that "*in burning fevers, a "yellowness of skin appearing on the fifth day, and accompanied by "hiccough, is a fatal symptom.*" * This is a very brief, exact, and appropriate description of the disease. A greater number are said to die of yellow fever on the sixth than any other day of the disease; and it very frequently happens that appearances of yellowness are discovered on the fifth, which, at that period, and accompanied by hiccough, constitute a fatal symptom. When the description which Hippocrates

Hippocrates gives of *Causus*, or *Burning Fever*, is duly recollected, and there is connected with this fever the occurrence of yellow skin, accompanied with hiccough, on the fifth day; a character results, which can apply to no other disease in the world but yellow fever.* And it would be exceedingly difficult, in so few words, to present a more expressive delineation of that distemper.

The terrible symptom of *black vomiting* is also frequently mentioned by Hippocrates, and represented as being of fatal import. He uses the phrases μελαινα χολη black bile, μελανα εμετον black vomit, and μελανων εμετον the vomiting of black matter. In the twelfth section of his Prognostics, he asserts, that if the matter vomited be of a livid or black colour, it betokens ill. In the first section of the first book of his *Coan Prognostics*, he enumerates black vomiting in a catalogue of the most fatal symptoms. And also in the fourth section of the same book, he considers porraceous, livid, or black vomiting, as indications of great malignancy.†

The importance of this conclusion is further illustrated and confirmed by adverting to the well known fact, that Hippocrates practised physic for a considerable portion of his life, in parts of Greece situated nearly in the same parallel of latitude with those in the United States, where the yellow fever has produced its greatest ravages.

*See Medical Repository, Hex. II. Vol.* 3, *page* 107.

On another account, the writings of Hippocrates offer important instruction concerning malignant fevers. Not the least reference to *contagion* is to be found in any part of them. If personal intercourse between the sick and the well had been the means of spreading these fevers from one individual or from one family to another, it is incredible that so prominent and glaring a fact should have escaped the notice of a person endowed with such talents for extensive, accurate, and discriminating observation.

### *Yellow Fever indigenous in the Island of Minorca.*

By the following quotation from *Cleghorn's Observations on the Epidemical Diseases of Minorca, from the year* 1744 *to* 1749, page 175 & 176, it appears that yellow fever often prevailed in that island more than sixty years ago, and that it was by no means considered as a new or extraordinary disease. It also appears, that the characteristic symptoms of yellow fever are often superinduced on the intermittent fevers of that place, and that their common tertian fevers are only a lower grade of yellow fever. The island of Minorca is fituated nearly in our latitude.

" But

---

* For the sake of removing all doubt on this subject, it is proper to submit the original to the reader's consideration:—

Εν τοισι καυσοισιν εαν επιγενηται ικτερος και λυξη πεμπταια εοντι, θανατωδες υποστροφαι λαμβανονται.

"But the utmost danger is to be apprehended, if a few drops of "blood fall from the nose: if black matter like the grounds of coffee, "is discharged upwards or downwards: if the urine is of a dark "hue and a strong offensive smell: if the whole skin is tinged with "a deep yellow, or any where discoloured with livid spots or suffu-"sions: if a cadaverous smell is perceptible about the patient's "bed: if in the time of the fit he continues cold and chilly, with-"out being able to recover heat; or if he becomes extremely hot, "speechless and stupid; has frequent sighs, groans, or hiccoughs; "and lies constantly on his back, with a ghastly countenance, his "eyes half shut, his mouth open, his belly swelled to an enormous "size, with an obstinate costiveness, or an involuntary discharge of "excrements: which formidable symptoms, as they seldom appear "before the third revolution of the disease, so they frequently "come on, both in double and simple intermittents, during the "fourth, fifth, or sixth period, even where the smallest danger was "not foreseen." The author likewise adds, in a note, that "The "English in Minorca are more liable than the natives to become "yellow in these fevers."

---

*On Yellow Fever in the interior of the Country.*

Sporadic cases of this disease are occasionally observed in all parts of the country. They are found more frequently and in greater number in low and marshy districts, near lakes, mill-ponds, swamps, &c. The most respectable physicians in the country so universally concur in this observation, that it would be unreasonable to contest the fact.

In some of the more exposed situations, and after very hot and damp summers, the yellow fever often assumes an epidemic appearance in the country. The malignant disease at Catskill in this State, in the year 1803, (see *Medical Repository*, vol. 8, page 105) affords an instance of this kind. In the year 1793, it prevailed in many parts of the country in the eastern, middle and southern States, where no suspicion of contagion could exist.

Dr. Anthon, of this city, whose accurate acquaintance with the pestilential epidemics of New York enables him to decide in the most satisfactory manner, assures me he has often seen the same disease in the interior country, and particularly in the low situations near the river *Illinois*, after an extensive inundation of that river, succeeded by hot weather.

Mr. Volney found yellow fever in several parts of the interior western country, during his travels in America, and describes the disease with so much accuracy and force, that no doubt of his testimony can be entertained.

*See his View of the Climate and Soil of the United States.*

Out of a great mass of particular instances of the appearances of yellow fever in situations inaccessible to foreign contagion, I shall only now select the following:

Extract

*Extract from Mr. Andrew Ellicott's Voyage down the River Ohio, in the month of November, 1796.*

"*November* 15*th.*

"Arrived at Galliopolis about 11 o'clock in the morning.—This village is a few miles below the mouth of the Great Kanhaway, on the west side of the Ohio river, and situated on a high bank; it is inhabited by a number of miserable French families. Many of the inhabitants, this season, fell victims to the yellow fever. The mortal cases were generally attended with the black vomiting. This disorder certainly originated in the town, and, in all probability, from the filthiness of the inhabitants, added to an unusual quantity of animal and vegetable putrefaction in a number of small ponds and marshes within the village.

"The fever could not have been taken there from the Atlantic States, as my boat was the first that descended the river after the fall of the waters in the spring: neither could it have been taken from New-Orleans, as there is no communication, at that season of the year, up the river, from the latter to the former of those places: moreover, the distance is so great, that a boat would not have time to ascend the river, after the disorder appeared that year in New-Orleans, before the winter would set in."

*See Ellicott's Journal.*

*The following fact is communicated by Dr. Watkins, from his personal knowledge.*

There is a village called *New-Design*, about fifteen miles from the Mississippi, and twenty miles from St. Louis, containing about forty houses and two hundred souls. It is on high ground, but surrounded by ponds. In 1797, the yellow fever carried off fifty-seven of the inhabitants, or more than a fourth. No person had arrived at that village from any part of the country where this fever had prevailed, for more than twelve months preceding. Our informant resided in the village at the time; and, having seen the disease in Philadelphia, he declares it to be the same that prevailed at New-Design. He also mentions an Indian village depopulated by the same disease two or three years before.

*See Medical Repository, vol. 4, page 74.*

*Fever, with black vomiting, in the middle part of Pennsylvania, west of the Susquehannah.*

"The fever which prevailed, in the autumn and winter of 1799, in Nittany and Bald-Eagle Valley, in Mifflin county, Pennsylvania, proved, in a number of cases, mortal. Bald-Eagle Valley, situated about 200 miles N. N. W. of Philadelphia, is low, abounding with much stagnated water in ponds, which, from the dryness of the season, became very putrid and offensive to the smell. Near to these waters the fever prevailed with great malignity. It was ushered in by chills, with pains in the back, limbs and head, which, in 48 or 60 hours, carried off the patients.—

They

They discharged vast quantities of filth from the stomach, of the consistence and appearance of coffee-grounds, so offensive in smell as to produce nausea, and even vomiting, in the attendants. The fæces also had the same appearance. In many the disease terminated by profuse discharges of blood from the anus and vagina.

*Ibid. page 75.*

---

*On* Dr. Chisholm's *singular opinions concerning Yellow Fever.*

It is well known that this gentleman contends for the production of a *new* and *peculiar pestilential disease*, which he supposes to have been imported by the *ship Hankey*, in the year 1793, from Boullam, on the coast of Africa.* He believes this new distemper to have been spread through the W. India islands and transmitted to this country. He admits that the yellew fever of the West-Indies, is not a contagious disease. The importers and contagionists in the United States, assuming his opinion, and fortifying themselves by his authority, assert that our epidemics are not the yellow fever of the West-Indies, but a continuation of the new and peculiar Boullam fever.

But the slightest examination of the subject is sufficient to satisfy an impartial inquirer, that the Boullam fever of Dr. Chisholm and the yellow fever of the West-Indies, are precisely the same disease; and that only such occasional variations of grade have been observed in it, as are found in the different epidemic seasons of all pestilential distempers. The ravages of pestilence in the West-Indies, since the pretended introduction of the Boullam disease, among a given number of Europeans or other strangers recently arrived, or among the natives themselves, are not greater than they were fifty years ago, or during the war of the American Revolution. The great body of physicians and people in the West-Indies, do not find the fever now prevailing at all different from what it was many years before the arrival of the ship Hankey from Boullam. The description of the disease by physicians who wrote forty, fifty, and sixty years ago, precisely agree with what is now observed in those islands and on this continent. And in this city, the yellow fever prevailed in the autumn of 1791, two years before the supposed arrival of the Boullam disease by the ship Hankey.

Without recurring, however, to facts of this kind, Dr. Chisholm's doctrine, considered in itself, cannot stand the contest of examination. All his leading assertions concerning the pretended introduction of the Boullam fever into the West-Indies, are positively denied by Mr. Paiba, a gentleman of intelligence and unblemished character, who was on board the ship charged with the importation,

* An Essay on the Malignant Pestilential Fever, &c. 2d Edit. in 2 vols.

portation, during the whole of the voyage. The narrative itself of the voyage, and of the disease supposed to have been imported, betrays inherent evidence of mistake. And even if Dr. Chisholm's story be admitted, it is only an instance of malignant disease generated in a vessel, as he does not pretend to derive it from the Africans.

Dr. Chisholm makes a very elaborate attempt to discriminate the features of the Boullam fever from those of the yellow fever of the West-Indies. It is apparent that there is no foundation for the distinction; and that he only describes different grades of the same disease, modified and rendered more malignant at one time than another, by peculiarities of season. This happens with respect to all epidemic diseases. The measles, for example, in one season, are *mild and safe;* at another, they are *malignant and fatal*; in one epidemic they are *highly inflammatory*, in another they may be *highly putrid*; yet are they not essentially the same disease? But, admitting, for argument's sake, the distinction contended for by Dr. C. it may be still asserted that, in his description of the ordinary yellow fever of the West-Indies, and not in that of the Boullam fever, he gives the character of the disease which has so often prevailed in this city.

It is creditable to the candour of Dr. Chisholm that he seems lately, in a considerable degree, at least in effect, to have given up his favourite opinion. He now admits that a disease, similar to that of Boullam, has been since generated on board of a filthy ship from England. It is proper to give his own words, as expressed in an extract of a letter to Dr. Davidson, dated Demarara, August 10, 1800, a period of seven years after the formation of his first opinion.

" A fever of the most alarming nature has most fatally prevailed since the beginning of July. I have visited a few of the sick at the resquest of Doctors Dunkin and Lloyd in town, and of Dr. Ord on this coast; and I have no hesitation in pronouncing it a fever of infection. Its features are, almost without exception, precisely those of the malignant pestilential fever of Grenada of 1793 and 1794. It is fully as fatal, as rapid, and as insidious. Its origin, as far as it has been ascertained by the gentlemen I have mentioned, seems to be similar. A ship arrived about the beginning of July or end of June from Liverpool, after touching at Surinam. The filth on board, occasioned by a cargo of horses, and the extreme neglect of the officers and crew, was such as beggars description."

*See Medical Repository, vol. 5, page 229.*

These facts, thus presented by Dr. Chisholm himself, form a luminous and instructive commentary on his former opinion, which he had published with great confidence, and which has been implicitly adopted and acted on by the contagionists in the United States. In 1793, he pronounced the malignant disease at Grenada, which, as was observed before, he believed to have been imported from the the coast of Africa, a "*nova pestis*," a peculiar, original, foreign pestilence, recently generated and utterly unknown before, endued with a new and distinct character. possessing new powers of devastation, and capable of propagating itself by contagion, throughout the world. As he considered it to have been engendered on board of the Hankey, in consequence of the accumulation of filth, the crowding of a great number of persons within a small space, and the heat of the atmosphere in which the vessel was immersed; he must have ascribed whatever peculiarity he supposed it to possess, to the peculiar state of the air on the coast of Africa; for he did not pretend to derive it originally from the inhabitants of Africa, or any modification of contagion. No other circumstance of the case, therefore, except some unknown singularity of the African atmosphere, could occasion this alleged instance of the generation of pestilence in a ship to differ from other cases in which malignant fevers are produced in filthy, crowded, and unventilated vessels, in hot climates or during hot seasons. But in the year 1800, while the flames of the Boullam disease lighted up in 1793, were still raging far and wide, and destroying the people of the West-Indies and of the American continent, he finds another "*nova pestis*," generated in a ship from England, which had touched at Surinam, and had become very filthy from a cargo of horses; and what is wonderful, he finds this pestilence, thus originating in a ship from England, possessing *features almost without exception, precisely those of the malignant, pestilential fever of Grenada, of 1793 and 1794; fully as fatal, as rapid, and as insidious.*—It appears then that the facts advanced by Dr. C. in the latter case (even admitting those concerning the Hankey to be true) instead of supporting his doctrine of *novelty and peculiarity in the fever of Boullam*, go too far for his purpose, and establish the general principle, that filthy, crowded, and unventilated vessels, immersed in a certain degree of heat and dampness, may generate malignant fever in all parts of the world where such circumstances are found,—which is precisely the principle for which the advocates of local and domestic origin have always contended.

As to Dr. C's opinion of the contagiousness of these fevers, it rests upon the same vague and delusive foundation with the popular, or rather vulgar inference of contagion, in all cases where a disease attacks a great number of persons in the same vicinity; which has been sufficiently refuted in a former part of this Report.

